# Shaping the Effects of Associative Brain Stimulation by Contractions of the Opposite Limb

**DOI:** 10.3389/fpsyg.2018.02249

**Published:** 2018-11-19

**Authors:** Richard G. Carson, Michelle L. Rankin

**Affiliations:** ^1^Trinity College Institute of Neuroscience and School of Psychology, Trinity College Dublin, Dublin, Ireland; ^2^School of Psychology, Queen’s University Belfast, Belfast, United Kingdom

**Keywords:** stroke, aging, paired associative stimulation, non-invasive brain stimulation, associative brain stimulation, corticospinal, primary motor cortex, contractions

## Abstract

There has been an explosion of interest in methods that may promote neural plasticity by indirectly stimulating tissue in damaged brains using transient magnetic fields or weak electrical currents. A major limitation of these approaches is that the induced variations in brain activity tend to be diffuse. Thus far it has proved extremely difficult to target pathways from the brain to specific muscles. This is a particular challenge for applications in rehabilitation. Stroke survivors often exhibit abnormal patterns of muscle activation, including diminished specificity and high levels of co-contraction. For the clinical relevance of brain stimulation to be enhanced, it is desirable that the effects can be restricted to pathways controlling muscles that are the specific targets of movement therapy. We have demonstrated previously that increases in the excitability of corticospinal projections to forearm muscles generated by paired associative stimulation (PAS), are modulated by contractions ipsilateral to the site of the cortical stimulus. The current aim was to determine whether in chronic stroke survivors, simultaneous contractions performed by the non-paretic limb increase the muscle specificity of changes in the excitability of projections to the impaired limb induced by PAS. Ten chronic stroke survivors, 13 age-equivalent and 27 younger healthy controls, completed two separate sessions/conditions. In one (PAS+CONT), isometric wrist flexion contractions of the non-impaired limb were made simultaneously with PAS. In the other (PAS), associative stimulation only was applied. In all groups, PAS alone gave rise to large increases in the excitability of projections to a wrist extensor muscle (extensor carpi radialis – ECR) that was not the target of stimulation. In marked contrast, for the stroke survivors, following combined PAS and flexion contractions of the non-impaired limb, there was no corresponding elevation in the excitability of corticospinal projections to the ECR of the paretic limb. A similar effect was present for the healthy young adults, but not expressed clearly for the age-equivalent controls. The implications of these findings with respect to the clinical deployment of non-invasive brain stimulation in movement rehabilitation are discussed.

## Introduction

Worldwide, there is an increasing incidence of stroke – a prototypical disorder of aging which affects 15 million people each year (WHO). National stroke strategies tend to emphasize the requirement for specialized therapeutic assistance in the months and years following stroke. To date, however, at least 40% of stroke survivors are left with significant residual impairments of the upper limb (Parker et al., 1986). As a consequence of financial constraints that limit the duration of acute hospital care, the therapeutic opportunities that are presented in the interval immediately following stroke are strictly limited. The quest for effective and efficient strategies of rehabilitation that will maximize the degree of recovery over the extended period following medical discharge has therefore become more imperative. It is now acknowledged that conventional rehabilitation techniques must therefore be augmented by new technologies and novel modes of therapy (Department of Health, 2007).

In recent years there has been an explosion of interest in non-invasive methods that promote neural plasticity by indirectly stimulating tissue in damaged regions of the brain using weak electrical currents or transient magnetic fields [e.g., transcranial magnetic stimulation (TMS)]. A major limitation is that the induced variations in brain activity tend to be diffuse. Thus far it has proved extremely challenging to target pathways from the brain to specific muscles. This is a particular concern in relation to the application of these methods to the rehabilitation of movement function following stroke. Stroke survivors often present with abnormal patterns of muscle activation including low specificity and high levels of muscle co-contraction (Dewald et al., 1995). For the clinical relevance of non-invasive brain stimulation (NIBS) to be enhanced, it would be advantageous to restrict its effects to pathways that control the muscles that are the specific targets of movement therapy.

Paired associative stimulation (PAS) has come to prominence not only as an experimental method with which to investigate Hebbian principles of neural plasticity, but also as a therapeutic intervention with the potential to treat brain injury/disease (e.g., Jayaram and Stinear, 2008; Castel-Lacanal et al., 2009; Palmer et al., 2018). We have demonstrated previously (Kennedy and Carson, 2008) that in young healthy individuals, elevations in the excitability of corticospinal projections to the forearm muscles brought about PAS, are modulated by the contraction of muscles ipsilateral to the site of cortical stimulation. Specifically, focal contractions (e.g., of the wrist flexors) applied by the opposite limb increase the muscle specificity of the induced increases in corticospinal excitability – restricting the effects of the associative stimulation to homologs of the principal muscles engaged in the contraction. In the present project, we sought to determine the clinical potential of this approach, by establishing whether in chronic stroke survivors, simultaneous contractions performed by the non-paretic limb increase the muscle specificity of changes in the excitability of corticospinal projections to the impaired limb induced by PAS.

It has been customary when examining the changes in excitability induced by NIBS to assess projections to the muscles of the hand. While these are studied conveniently in the laboratory, they are not necessarily representative of the muscles that are the targets of rehabilitation. A substantial proportion of stroke survivors do not have the facility to generate functional movements using the muscles of the shoulder and the upper arm, let alone individuated movements of the fingers. For this reason we focus our investigations on induced changes in the excitability of corticospinal projections to muscles of the forearm [typically flexor carpi radialis (FCR) and extensor carpi radialis (ECR)]. In this context, we utilized a variant of PAS described by Castel-Lacanal et al. (2007), whereby the peripheral nerve stimulation component of the associative protocol consists of a train of six pulses delivered at 10 Hz (see also Ridding and Taylor, 2001) applied over the motor point of the target muscle, rather than a single stimulus delivered to the nerve trunk. In a previous investigation using this technique, we were able to induce increases in corticospinal excitability that persisted for more than 30 min following the cessation of stimulation (Carson et al., 2013). As electrical stimulation over the motor point can be accomplished more readily than stimulation of a peripheral nerve trunk, this variant has greater potential for clinical application. In addition, it permits the stimulation of muscles for which the trunk of the innervating nerve is not accessible.

## Materials and Methods

### Participants

Twenty-seven young healthy volunteers, 10 chronic stroke survivors, and 13 “age equivalent” older healthy volunteers participated in this study. The demographic characteristics of the groups are presented in Table 1. The study was reviewed and approved by the Queen’s University Belfast School of Psychology Ethics Committee. All participants gave written informed consent, in accordance with the Declaration of Helsinki.

**Table 1:** Participant characteristics.

	Young (*n* = 27)	Older (*n* = 13)	Stroke survivors (*n* = 10)
Age (mean years)	23 (*SD* = 4)	68 (*SD* = 5)	62 (*SD* = 14)
Age (median years)	22	68	67
Male/female	12/15	4/9	9/1
Time since stroke (years)			3.8 (*SD* = 4)
Arm tested (left/right)	13/14	6/7	7/3
Site of lesion (C/SC/C+SC)			3/5/2
FMA-UE score (max = 66)			54 (*SD* = 6)

*The age, time elapsed since stoke, and the Fugl-Meyer Assessment of the Upper Extremity (FMA-UE) score, are shown as the mean plus standard deviation. With respect to the site of lesion, the codes are as follows: C+SC (cortical and sub-cortical); C (cortical); SC (sub-cortical).*

The recruitment of chronic stroke survivors was through community advertising. We compiled a database of 104 chronic stroke survivors, who expressed an interest in participating in the research. Of this group, 61 individuals were excluded after a follow up phone call, during which it was stated that they were left handed, contraindications became apparent, or they elected not to participate. The remaining 43 people were visited in their homes, in order to undertake two screening procedures: the Star Cancellation Test (for spatial neglect) and the Hodkinson Mental Test (for cognitive function). These individuals also completed a general medical history questionnaire that was assessed by both the first author and a consultant gerontologist. None were engaged in rehabilitation therapy during the course of the study. Inclusion criteria were (1) >6 months post-stroke; (2) no diagnosed cognitive impairment or spatial neglect; (3) no contraindications to TMS; (4) some voluntary control of shoulder/elbow/wrist movement; (5) originally right-handed. The 15 people deemed suitable for participation in the laboratory-based procedures were assessed using standard clinical measures of motor function [Fugl-Meyer Assessment of the Upper Extremity (FMA-UE)]. Of this group, four individuals failed to exhibit motor evoked potentials (MEPs) in the muscles of their affected arm, and one person was unable to maintain their muscles in a relaxed state during the laboratory testing procedures. Ten individuals were therefore included in the complete study. Of these, nine underwent MR scans (Axial T2; Axial diffusion weighted imaging; Axial T1), which were read by a consultant neuroradiologist. A CT scan was used as the basis for classification of the remaining individual. For seven of the stroke survivors, their left arm was more affected. For the other three, their right arm was more affected. In all cases the affected (i.e., impaired) arm was designated the “target” arm. For all of the stroke survivors their right hand had been their preferred hand.

The healthy volunteers were right handed according to the Edinburgh handedness inventory (Oldfield, 1971). For 13 of the 27 young volunteers, the left arm was designated the target. For six of the 13 older volunteers, the left arm was designated the target. The opposite limb was designated the “active arm.”

### Recording and Stimulation Procedures

The participants were seated with the upper limbs supported and stabilized by vacuum cushions, the forearms in mid-pronation and the elbows semi-flexed (100–120°). The left and right hands were secured in manipulanda mounted on torque transducers located coaxially with the (flexion–extension) axes of rotation of the wrists. Electromyographic (EMG) activity was recorded from the ECR longus and the FCR muscles of both arms, using pairs of silver chloride (AgCl) electrodes. For a given electrode pair, the centers were separated by approximately 3 cm. The position of the electrode pair over ECR longus was in accordance with the procedure described by Riek et al. (2000). The FCR recording electrodes were placed adjacent to the motor point. EMG signals were amplified (gain = 1000), bandpass filtered (20–2,000 Hz), and digitized (16 bit) at a sampling rate of 5 kHz.

Magnetic stimuli were delivered to the primary motor cortex (M1) contralateral to the target limb by a Magstim 200 stimulator (Magstim Company, Whitland, Dyfed, United Kingdom), using a figure of eight coil (internal wing diameter 55 mm), located at the optimal position (“hot spot”) to obtain a MEP in the FCR muscle. The coil was placed so that the axis of intersection between the two loops was oriented at approximately 45° to the sagittal plane, to induce posterior to anterior current flow across the motor strip. The hot spot having been established, the lowest stimulation intensity at which MEPs with peak-to-peak amplitude of approximately 50 μV were evoked in at least 5 of 10 consecutive trials was determined to be the resting motor threshold (rMT). The level of stimulation used during the experiment was 120% of the FCR rMT. At this intensity a motor potential was also evoked in the ECR of the target arm.

Paired associative stimulation was conducted following Castel-Lacanal et al. (2007). This consisted of electrical stimulation applied to the motor point of the target FCR muscle, paired with a single TMS pulse applied to the contralateral hemisphere. The electrical stimulation consisted of a train of six 1 ms pulses delivered at a frequency of 10 Hz (i.e., 500 ms interval between the first and sixth pulses), generated by a constant current square wave stimulator (Grass S88; Grass Technologies, Rhode Island, United States). The intensity of the electrical stimulation was that which elicited a minimal motor response in the target FCR, and resulted in just visible motion of the tendon at the level of the wrist.

The voltage signal corresponding to flexion torque applied at the wrist by the “active arm” was displayed on a computer screen. This was placed directly in front of the participants at eye level. The signal was portrayed as a filled white bar superimposed upon a graduated background consisting of white horizontal lines. The height of the bar corresponded to the magnitude of the applied torque. In each case, this was normalized with respect to the mean torque generated by the participant during three maximum voluntary contractions (MVC) performed at the commencement of the experiment. At the start of each stimulus epoch, a horizontal line corresponding to the target level of torque (20% MVC) changed from white to red. The participant was asked to generate an increasing level of wrist flexion torque such that the filled white bar reached the target line. When the applied torque was within the target zone (i.e., ±2% of the required value), and maintained there for 100 ms, an external trigger signal was generated.

### Experimental Protocol

Paired associative stimulation consisted of a train applied to the motor point of the target FCR muscle, followed 25 ms after the last stimulus of the train by a single pulse of TMS delivered to the motor hotspot of the target FCR. This was repeated every 10 s for a period of 30 min. Two conditions were employed in this experiment: PAS plus contraction (PAS + CONT) and PAS only (PAS). The order of allocation to conditions was counterbalanced across participants. Successive testing sessions were separated by at least 7 days (mean 24 days). For each participant, all sessions commenced at the same time of day – in order to control for any potential influence of circadian rhythms (Sale et al., 2007).

Each session commenced with three blocks of pre-intervention measurements. During each of these no contractions were performed. In each block, 10 MEPs were generated at intervals ranging between 4 and 8 s. Successive blocks of measurements started at intervals of 2 min.

The intervention then commenced. In the PAS plus contraction condition, a target level of active arm wrist flexion torque corresponding to 20% MVC was displayed on the computer screen. Once the target level of torque was achieved, the external trigger caused the participant to receive electrical stimulation of the target flexor motor point with TMS delivered to the contralateral M1 (i.e., PAS) 25 ms after the last stimulus in the train. This was repeated at 10 s intervals for 30 min, yielding a total of 180 stimulus pairs. In the PAS only condition, the target level of torque was set to zero. In this instance, stimulus delivery was triggered without the requirement for wrist flexion torque. As in the PAS plus contraction condition, paired stimulation was delivered every 10 s for 30 min (180 stimulus pairs in total).

Commencing less than 1 min following completion of the intervention, three blocks of post-intervention measurements were undertaken. During these measurements there were no contractions. As was the case for the pre-intervention trials, in each block 10 MEPs were elicited, Following an interval of 10 min after the start of the initial ‘post’ blocks, a further 30 MEPs were obtained (in three blocks). These were referred to as ‘post ten.’ A further set of 30 MEPs was elicited 20 min following. These were referred to as “post-twenty.” The final post intervention measurements (‘post-thirty’) commenced 30 min following the start of the initial ‘post’ blocks.

### Data Analysis

The root mean square (rms) of the background EMG recorded in FCR and ECR was calculated for a window 93–3 ms before TMS onset. If the value was greater than 6 μV for either muscle, the corresponding MEP was excluded. As an additional basis upon which to eliminate instances in which elevated excitability of the spinal motoneuron pool may have influenced the MEP amplitude, we first calculated for each participant (separately for ECR and FCR) the quartiles for all background rms EMG values retained following the screening procedure described above. In the event that an individual rms value was above the upper whisker of the distribution (set to the third quartile plus 1.5 times the interquartile range) the corresponding MEP was excluded.

For the retained recordings, the trimmed (20%) mean (peak-to-peak) amplitude of the MEPs in each block was calculated. In light of recent analyses of within session MEP amplitude variability (Cavaleri et al., 2017), a minimum of five responses (i.e., following the screening procedures described above) in each block was required. For each time of measurement (Pre, Post0, Post10, Post20, and Post30), the mean of the values (i.e., the trimmed means) obtained for the three constituent blocks was taken as the dependent measure.

It is well known that the distributions of MEP amplitude values tend to exhibit substantial deviations from normality (Nielsen, 1996). In the present case, when the sample distributions of the dependent measure were assessed using the Shapiro–Wilks test, deviations from normality were the norm, rather than an exception. A Yeo-Johnson power transformation was therefore applied to each of the six sample distributions (i.e., 3 groups by 2 muscles). Following the transformation, there were no instances in which any of the 10 cells (2 experimental conditions × 5 times of measurement) in each sample, satisfied the conventional criterion (Shapiro–Wilks, *p* < 0.05) that indicates deviations from normality. The values shown in the figures (means and confidence intervals) were obtained by applying the inverse transforms.

Mixed effects models in which participant was a random effect, and condition (PAS, PAS plus contraction) and time (levels = Pre, Post0, Post10, Post20, and Post30) were fixed effects, were calculated separately for each muscle (FCR, ECR), and for each group of participants (young, stroke survivors, older) using the lmerTest package in R. In fitting the models, restricted maximum likelihood (REML) estimation and an unstructured covariance matrix were employed. On this basis, planned contrasts were conducted between the value obtained prior to the intervention (Pre), and the value calculated for each time point following the intervention (Post0, Post10, Post20, and Post30). Additional planned comparisons contrasted the value obtained prior to the intervention to a single pooled value derived for all four post-intervention measurements. The exact probabilities associated with each comparison are reported in Tables 1–3. The relevant degrees of freedom were obtained using Kenward-Roger’s approximation. In the case of balanced designs, such as those employed in the present study, this yields values equivalent to those of a repeated measures ANOVA design.

The modified Bonferroni adjustment for multiple comparisons (Keppel, 1991) was calculated. The aim of this procedure is to control for the potential elevation of family-wise error associated with multiple (planned) comparisons. The implicitly assumed family-wise error is obtained as the product of the degrees of freedom (*df*) and the alpha level (conventionally 0.05). In the event that the theoretically motivated set of planned comparisons exceeds the *df*, it is necessary to adjust the effective level of alpha in order to preserve the family-wise error rate. Given the relatively small number of planned comparisons conducted in the present study (i.e., relative to the relevant degrees of freedom), the application of this procedure resulted in the retention of an alpha level of 0.05.

In order to further assist in the interpretation of the tests of significance, in particular with a view to comparing the three groups of participants included in this study, the unbiased effect size index for ANOVA (*f*) (Cohen, 1988) was calculated for each planned contrast following Nakagawa and Cuthill (2007). This dimensionless index, describes the degree of departure from no effect. It can be viewed as the degree to which the phenomenon is manifested. A large effect size is considered by convention to be indicated by an *f* of 0.4, a medium effect size by an *f* of 0.25, and a small effect size by an *f* of 0.1.

**FIGURE 1:**
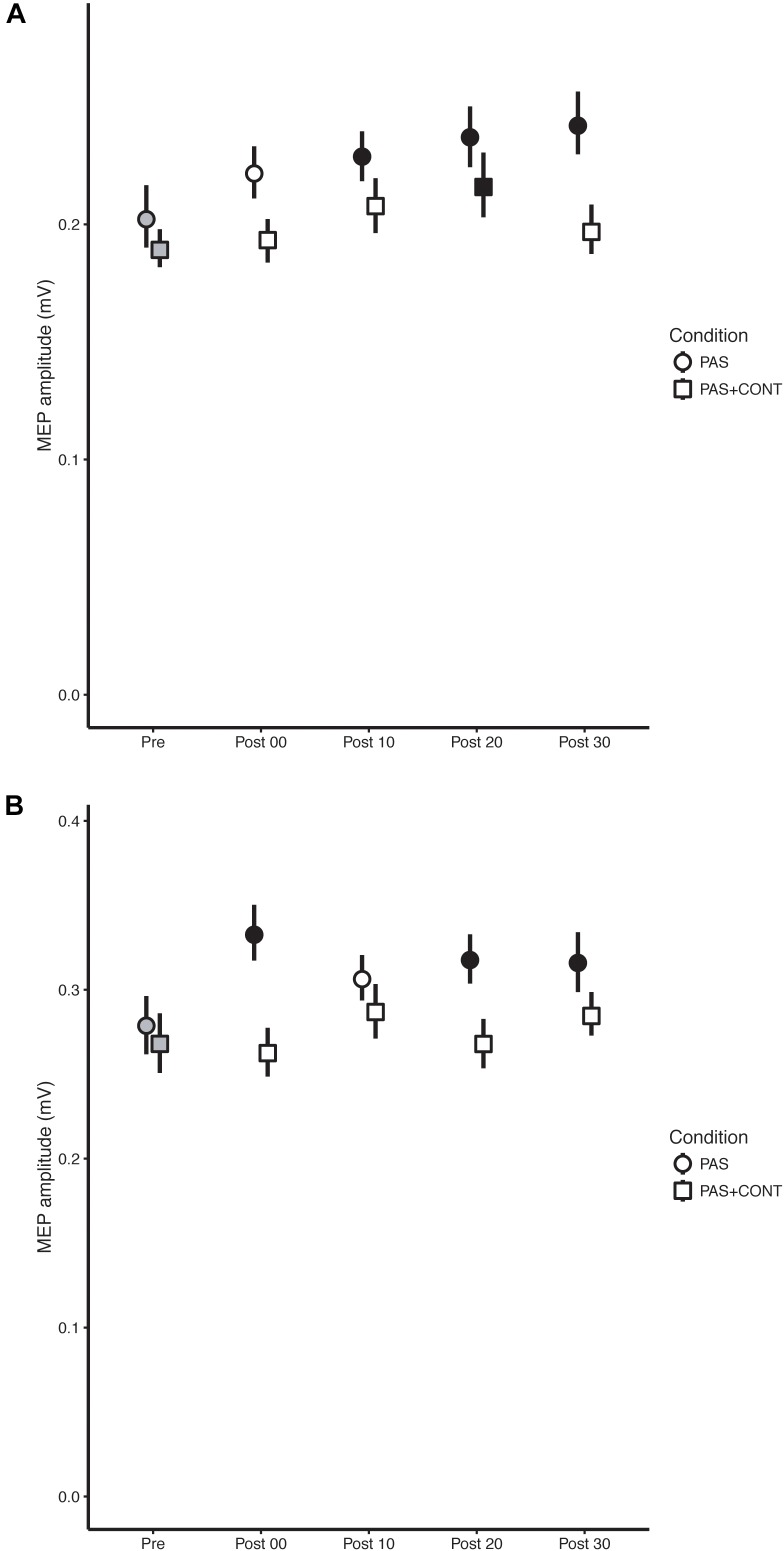
Young healthy participants. The mean (*n* = 27) MEP amplitude values obtained for the flexor carpi radialis (FCR) muscle **(A)** and extensor carpi radialis (ECR) muscle **(B)** at each time of measurement (Pre, Post00, Post10, Post20, and Post30), are shown for the respective experimental conditions: paired associative stimulation (PAS); PAS plus contraction (PAS + CONT). The error bars are the 95% “Cousineau-Morey” confidence intervals. The values recorded prior to the intervention are shown as filled gray symbols. Instances in which a post-intervention measurement differed reliably from the pre-intervention value are shown as filled black symbols.

**FIGURE 2:**
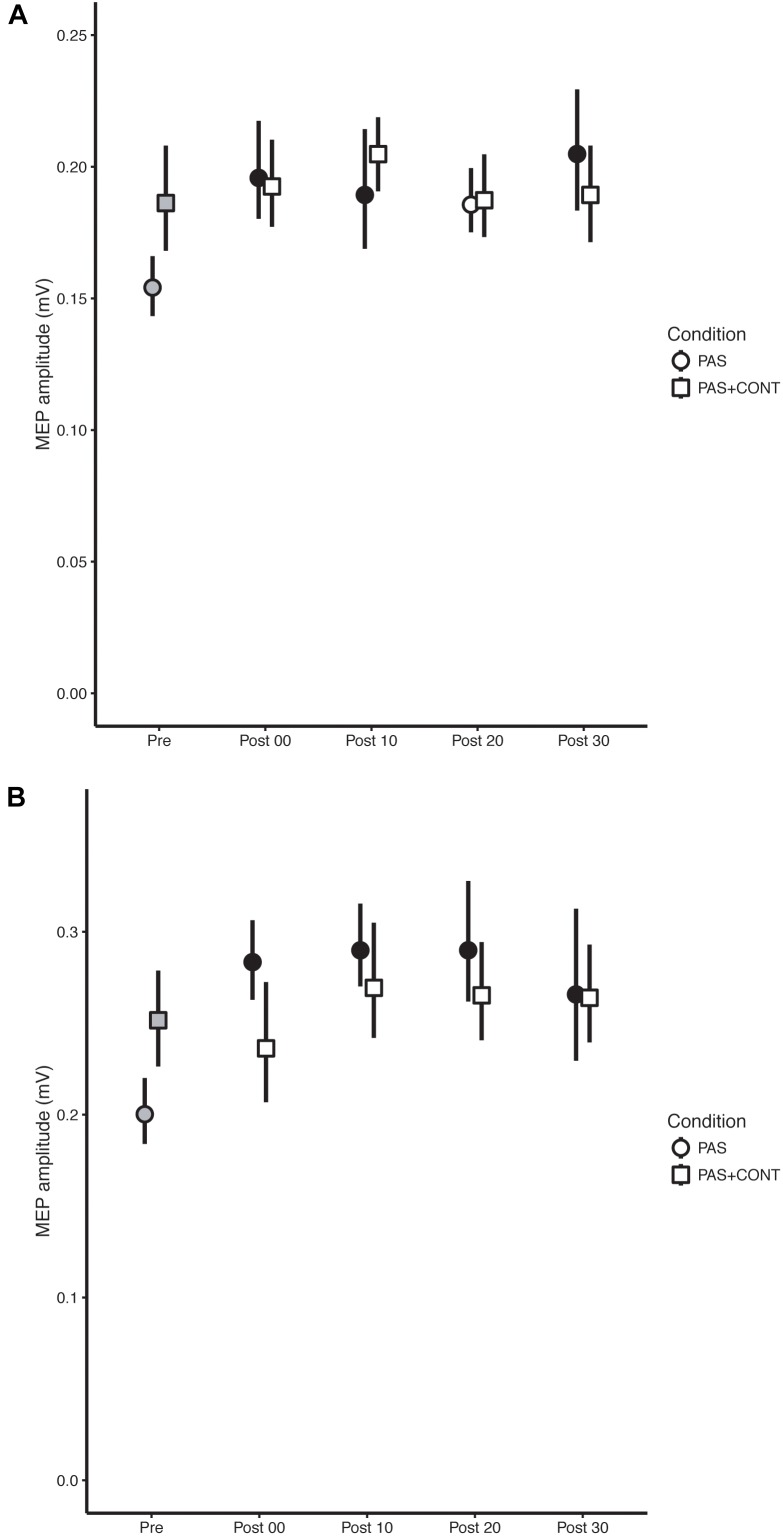
Stroke survivors. The mean (*n* = 10) MEP amplitude values obtained for the flexor carpi radialis (FCR) muscle **(A)** and extensor carpi radialis (ECR) muscle **(B)** at each time of measurement (Pre, Post00, Post10, Post20, and Post30), are shown for the respective experimental conditions: paired associative stimulation (PAS); PAS plus contraction (PAS + CONT). The error bars are the 95% “Cousineau-Morey” confidence intervals. The values recorded prior to the intervention are shown as filled gray symbols. Instances in which a post-intervention measurement differed reliably from the pre-intervention value are shown as filled black symbols.

**FIGURE 3:**
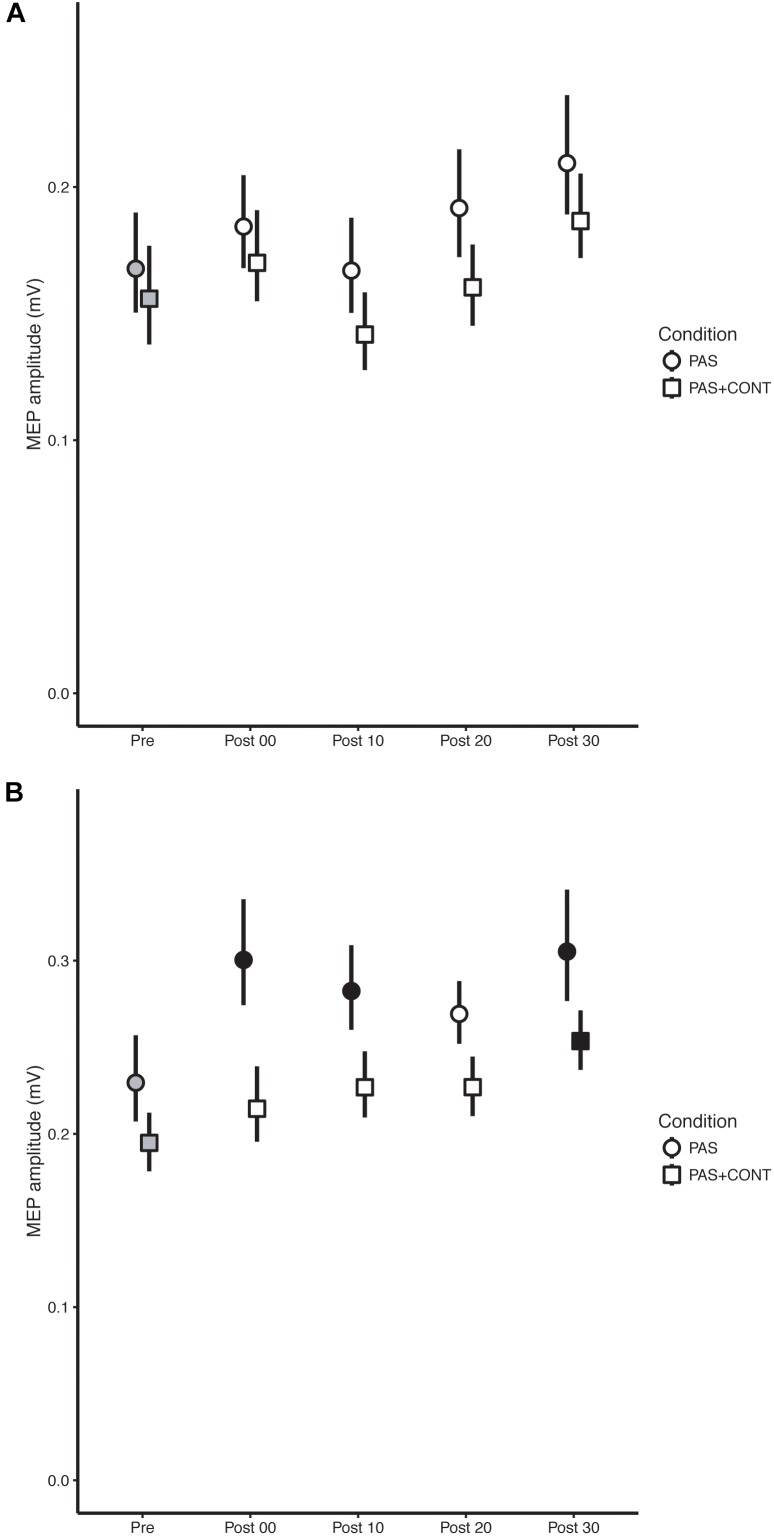
Older healthy participants. The mean (*n* = 13) MEP amplitude values obtained for the flexor carpi radialis (FCR) muscle **(A)** and extensor carpi radialis (ECR) muscle **(B)** at each time of measurement (Pre, Post00, Post10, Post20, Post30), are shown for the respective experimental conditions: paired associative stimulation (PAS); PAS plus contraction (PAS + CONT). The error bars are the 95% “Cousineau-Morey” confidence intervals. The values recorded prior to the intervention are shown as filled gray symbols. Instances in which a post-intervention measurement differed reliably from the pre-intervention value are shown as filled black symbols.

In order to show confidence intervals that accurately reflect the outcomes of the inferential statistics, i.e., tests of difference undertaken in the context of a repeated measures design, the error bars included on Figures 1–3 were obtained in accordance with Cousineau (2005) and Morey (2008). They were computed using the procedures described by Politzer-Ahles (2017).

## Results

### Young Healthy

In the PAS condition, MEPs recorded in FCR following the intervention were elevated relative to those obtained prior to the intervention (Table 2). This effect was also expressed reliably for the individual contrasts – with the exception of that based on the value obtained immediately followed the termination of PAS. With respect to the contrasts performed for the individual time points, the (unbiased) effect sizes were typically of small to medium size (*f* = 0.14–0.29) (Table 2). In the PAS + contraction condition, a reliable elevation in MEP amplitude was observed only at 20 min post intervention (Figure 1A).

**Table 2:** Young healthy participants: *F* ratios, *p*-values and (unbiased) effect sizes for comparisons between the MEP amplitudes obtained prior to the intervention (Pre), and (i) the pooled values obtained across all time points following the intervention, and (ii) the values obtained at each of four time points following the intervention.

Condition	Pre vs.	*F*(1,104)	*p*-Value	Effect size (*f*)
**FCR**				
PAS	All post	8.02	0.005	0.28
	Post00	2.22	0.139	0.14
	Post10	4.09	0.045	0.20
	Post20	6.87	0.010	0.25
	Post30	8.86	0.004	0.29
PAS + CONT	All post	2.10	0.148	0.14
	Post00	0.12	0.731	0.03
	Post10	2.28	0.134	0.15
	Post20	4.60	0.034	0.21
	Post30	0.40	0.529	0.06
**ECR**				
PAS	All post	10.67	0.001	0.32
	Post00	12.87	<0.001	0.34
	Post10	3.47	0. 069	0.18
	Post20	6.79	0.010	0.25
	Post30	6.22	0.014	0.24
PAS + CONT	All post	0.43	0.514	0.06
	Post00	0.16	0.693	0.04
	Post10	1.75	0.189	0.12
	Post20	0.00	0.996	0.00
	Post30	1.35	0.248	0.11

*The results for the flexor carpi radialis (FCR) and extensor carpi radialis (ECR) are given separately.*

Paired associative stimulation gave rise to pronounced elevation (*f* = 0.18–0.35) in the amplitude of MEPs recorded in the ECR muscle. In marked contrast, in the PAS + contraction condition, the amplitudes of MEPs obtained following the intervention could not be distinguished from those recorded prior to the intervention (Figure 1B).

### Stroke Survivors

In the PAS condition, MEPs recorded in FCR following the intervention were elevated relative to those obtained prior to the intervention (Figure 2A). With respect to the contrasts performed for individual time points, the (unbiased) effect sizes were in the range of medium to large (*f* = 0.32–0.47) (Table 3). In the PAS + contraction condition, the amplitudes of MEPs obtained following the intervention could not be distinguished from those recorded prior to the intervention (Figure 2A).

**Table 3:** Stroke survivors: *F* ratios, *p*-values and (unbiased) effect sizes for comparisons between the MEP amplitudes obtained prior to the intervention (Pre), and (i) the pooled values obtained across all time points following the intervention, and (ii) the values obtained at each of four time points following the intervention.

Condition	Pre vs.	*F*(1,31)	*p*-Value	Effect size (*f*)
**FCR**				
PAS	All post	7.97	0.006	0.51
	Post00	7.37	0.011	0.46
	Post10	4.17	0.049	0.35
	Post20	3.42	0.074	0.32
	Post30	7.56	0.010	0.47
PAS + CONT	All post	0.03	0.868	0.03
	Post00	0.10	0.758	0.05
	Post10	0.25	0.618	0.09
	Post20	0.03	0.862	0.03
	Post30	0.01	0.936	0.01
**ECR**				
PAS	All post	17.88	<0.001	0.77
	Post00	16.56	<0.001	0.71
	Post10	13.79	<0.001	0.64
	Post20	13.99	<0.001	0.66
	Post30	10.18	0.003	0.55
PAS + CONT	All post	0.46	0.514	0.12
	Post00	0.44	0.510	0.12
	Post10	1.64	0.210	0.22
	Post20	0.60	0.444	0.13
	Post30	0.54	0.467	0.13

*The results for the flexor carpi radialis (FCR) and extensor carpi radialis (ECR) are given separately.*

Paired associative stimulation brought about extremely large (*f* = 0.55–0.71) (Table 3) increases in the amplitude of MEPs recorded in the ECR muscle (Figure 2B). Whereas, in the PAS + contraction condition, the amplitudes of the MEPs recorded in ECR following the intervention could not be distinguished reliably from those recorded prior to its onset (Figure 2B).

### Older Healthy

In neither the PAS nor the PAS + contraction condition were there clear indications that the intervention gave rise to reliable changes in the amplitudes of MEPs recorded in FCR (Figure 3A).

Although the degree to which the effect was expressed varied somewhat over the course of the interval following the cessation of the intervention (Table 4), in both the PAS and PAS + contraction conditions, the amplitudes of MEPs recorded in ECR following the intervention were larger than those obtained before it commenced (Figure 3B).

**Table 4:** Older healthy participants: *F* ratios, *p*-values and (unbiased) effect sizes for comparisons between the MEP amplitudes obtained prior to the intervention (Pre), and (i) the pooled values obtained across all time points following the intervention, and (ii) the values obtained at each of four time points following the intervention.

Condition	Pre vs.	*F*(1,47)	*p*-Value	Effect size (*f*)
**FCR**				
PAS	All post	1.25	0.267	0.16
	Post00	0.59	0.447	0.11
	Post10	0.00	0.964	0.01
	Post20	1.21	0.276	0.16
	Post30	3.55	0.066	0.27
PAS + CONT	All post	0.31	0.577	0.08
	Post00	0.59	0.448	0.11
	Post10	0.67	0.414	0.12
	Post20	0.06	0.806	0.03
	Post30	2.60	0.114	0.23
**ECR**				
PAS	All post	5.31	0.024	0.33
	Post00	4.93	0.032	0.31
	Post10	4.91	0.032	0.31
	Post20	0.72	0.401	0.12
	Post30	5.89	0.019	0.34
PAS + CONT	All post	6.39	0.013	0.37
	Post00	1.34	0.252	0.16
	Post10	3.48	0.068	0.26
	Post20	3.50	0.067	0.26
	Post30	11.53	0.001	0.48

*The results for the flexor carpi radialis (FCR) and extensor carpi radialis (ECR) are given separately.*

### Supplementary Analyses

As there are a number of methods other than the trimmed mean that can be used to calculate a measure of central tendency from a set of MEP amplitude measurements, two sets of supplementary analyses were undertaken. In the first, the median was used in place of the trimmed mean. In the second, the natural logarithm of each MEP amplitude value was employed. These logarithmic values were then averaged. The exponential of the average was used as the measure of central tendency. Inferential analyses conducted on the basis of these additional measures produced precisely the same pattern of outcomes as those based on the trimmed means, and effect sizes that were effectively equivalent.

## Discussion

It has frequently been contended that the effects of PAS exhibit “topographical specificity” (e.g., Morgante et al., 2006; Castel-Lacanal et al., 2007; Quartarone et al., 2008). This has been taken to imply that the changes in excitability that are brought about by the intervention are restricted to the cortical representations of muscles innervated by the peripheral nerve that was subject to electrical stimulation (Stefan et al., 2000). As we have discussed in detail elsewhere (Carson and Kennedy, 2013), the empirical origins of this supposition are not readily apparent. Indeed, there is an abundance of evidence to the contrary (see Carson and Kennedy, 2013). Castel-Lacanal et al. (2007) applied 500 ms (10 Hz) trains of electrical stimulation to the ECR motor point, followed 25 ms later by TMS to the contralateral M1. They reported increases in the magnitude of MEPs elicited in FCR (η^2^ = 0.26) that were of comparable magnitude to those registered for ECR (η^2^ = 0.27). In the present study, FCR was the target. In all three groups of participants, the magnitude of the increases in ECR MEP amplitude brought about by PAS alone were greater than the changes obtained for the FCR. Indeed, for the older healthy participants, the intervention failed to bring about reliable changes in the magnitude of MEPs recorded in FCR. What are the potential origins of these distributed effects?

There exists the possibility that the low intensity wrist flexion contractions brought about by the electrical stimulation (at the minimum intensity necessary to induce a visible displacement of the FCR tendon), were sufficient to increase the length of the ECR muscle to an extent that an ascending afferent volley was generated. As a consequence of electromechanical constraints, the corollary of any such volley would necessarily have been delayed relative to that induced directly by the electrical stimulation of the FCR motor point. This would not, however, have precluded interactions (e.g., in cortical networks) with the temporally extended physiological sequelae of TMS. On the basis of studies in animals, it is evident that a single magnetic stimulus gives rise to episodes of enhanced and suppressed single-unit activity (Mueller et al., 2014; Li et al., 2017) that, in the context of general facilitation, may persist for at least 500 ms (Moliadze et al., 2003). There are, however, reasons to believe that interactions arising from changes in the length of the ECR muscle brought about by contractions of FCR, are unlikely to have provided a basis for the observed outcomes. The effects induced by PAS when trains of electrical stimulation are applied to biceps brachii (innervated by the musculocutaneous nerve), extend to ECR (innervated by the radial nerve), and to FCR (innervated by the median nerve). When the FCR is the target of PAS, increases in the excitability of corticospinal projections to BB and ECR are obtained – in addition to those present for FCR (Carson et al., 2013). Since in these examples the effects of PAS encompass muscles located on another part of the limb, changes in their length brought about by contractions of the target muscle, were evidently not necessary for increases in corticospinal excitability to occur.

Since standard PAS protocols (including the one used in the present study) deliver TMS at intensities above motor threshold, changes in synaptic activity at a various levels of the neuraxis may be driven by re-afferent feedback arising from the ensuing muscle contractions (Lang et al., 2006; Carson and Kennedy, 2013). Importantly, the physiological effects of TMS are never restricted to the notional target muscle (i.e., for which the scalp “hotspot” has been obtained). Evoked potentials can be recorded from multiple muscles, often widely distributed and with heterogenous innervation. If the intensity of TMS is sufficient, contraction induced changes in the lengths of these muscles will occur. As we have highlighted elsewhere (Carson and Kennedy, 2013), the consequential reafferent volleys, although delayed by tens of milliseconds relative to the initial cortical stimulation, are maintained for hundreds of milliseconds thereafter. The extent of the neural activity induced in M1 by such reafference can be appreciably larger than that which is brought about by the direct sensory consequences of peripheral stimulation. Similarly, perhaps 10% of the BOLD signal change registered in M1 following suprathreshold TMS is induced by inputs from muscle afferents (Shitara et al., 2013). Aside from these reafference driven effects, both TMS and electrical stimulation of peripheral nerves produce complex spreading patterns of activation that can be registered throughout the brain for extended periods following the cessation of stimulation. As a consequence, neural circuits extending beyond the primary somatosensory – primary motor axis, encompassing cerebello-thalamo-cortical and thalmo-cortical pathways, exhibit the potential to mediate the changes in corticospinal excitability that are brought about by PAS (Carson and Kennedy, 2013). While the specific neural circuits underpinning the changes in corticospinal excitability brought about by such associative protocols remain to be elucidated, the mediating processes evidently do not exert effects that are confined to pathways projecting to muscles targeted by the peripheral nerve stimulation.

The origin of these distributed effects notwithstanding, the principle focus of the present study was upon the degree to which they might be restricted to specific muscles through focal contractions of the opposite limb. In accordance with our previous investigations, it was hypothesized that contractions of the opposite (“active”) limb that engaged wrist flexor muscles, would serve to limit the effects of PAS to a homologous muscle of the “target” limb. In our original study, which engaged only right-handed, young healthy participants, the right FCR was in all cases the target for PAS. Both 14 and 28 min of combined PAS and left wrist flexion contractions resulted in reliable increases in right FCR MEP amplitude, which were not present in right ECR (Kennedy and Carson, 2008). In the present study, combined PAS and (opposite limb wrist flexion) contractions failed to give rise to changes in the amplitude of FCR MEPs in most cases. In order to address the possibility that this difference in outcomes was accounted for the fact that half of the healthy participants in the present performed the contractions with their preferred right limb, two additional analyses were undertaken. In these, the cohort of young healthy participants was first divided in accordance with the limb used to perform the contractions (left or right). The outcomes of these analyses were not, however, materially different from those undertaken for the whole cohort – the PAS condition gave rise to increases in FCR MEP amplitude, whereas the PAS plus contraction condition did not.

In another respect the outcomes of the present study were in accordance with our hypothesis, and consistent with our previous observations. In young healthy participants, flexion contractions performed by the opposite limb served to eliminate increases in the amplitude of MEPs recorded in ECR that were otherwise brought about by PAS. This was also the case for the stroke survivors. Indeed, the general pattern of results for this group was in close accordance with that of the young healthy participants. Specifically, PAS gave rise to increases in the amplitude of MEPs recorded in FCR and ECR. In the PAS plus contraction condition, no such changes were observed.

While in the young healthy participants and the stroke survivors, wrist flexion contractions generated by the opposite limb had the anticipated effect of nullifying increases in the excitability of corticospinal projections to the wrist extensor (ECR), they also dramatically reduced the impact of the PAS upon the state of corticospinal projections to the target FCR muscle. What might account for the fact that the effect of the contractions upon the state of the corticospinal projections to FCR was different from that which we observed previously (Kennedy and Carson, 2008)?

In our original study, the PAS protocol comprised a single electrical stimulus delivered to the median nerve (at the elbow), at an intensity that elicited an H-wave of 100 μV in the target FCR muscle (≈ 2.2 times perceptual threshold). TMS was applied to the opposite M1 following an interval calculated separately for each participant, using a formula that utilized the MEP onset latency, and the peripheral nerve stimulation M-wave onset latency. The average inter-stimulus interval was 18.7 ms. In the PAS plus contraction condition therefore, the time that elapsed between the trigger generated when the wrist flexion contraction achieved the 20% MVC criterion (which immediately caused delivery of the single electrical stimulus to the median nerve), and the application of TMS to the opposite M1, was never greater than 25 ms. In the present study, as a consequence of 500 ms trains of electrical stimulation being used, the interval between the trigger generated by the wrist flexion contraction and the application of TMS was 525 ms in all cases. It is possible that the temporal disjunction between the triggering contraction and the ensuing cortical stimulation may have served to disrupt the associative effects that were otherwise realized (i.e., for FCR) by the PAS protocol. In particular, the cortical stimulation (and some elements of the nerve stimulation train) occurred in a period during which there was a reduction (if not an absence) of neural drive to the FCR of the active limb. Taken together, this suggests that the attenuating effect of the wrist flexion contractions (i.e., of the opposite limb) on the PAS induced effects otherwise observed for ECR, and the accentuating effects observed previously for FCR (i.e., Kennedy and Carson, 2008) may reflect at least partially distinct processes. The attenuating effects for ECR were observed when a PAS protocol based on extended trains of motor point stimulation was used in the present study, whereas accentuating effects for FCR were not apparent. In this regard, it is also worth noting that the corticospinal projections to FCR and ECR appear to be differentially sensitive to natural (e.g., change in muscle length during active movements, Chye et al., 2010) and artificial (muscle tendon vibration, Forner-Cordero et al., 2008) variations in the balance of efferent/afferent drive. Although, to the best of our knowledge, analogous effects have not been documented for PAS or for isometric contractions of the opposite limb, these previous findings reinforce the possibility that a multiplicity of mediating processes are likely to be implicated, and that these may differ in their expression across muscle groups.

Another, and perhaps more parsimonious, explanation is that the attenuating effect of the contractions performed by the opposite limb reflected a single process that had an equivalent influence upon the effects otherwise brought about by PAS on the state of corticospinal projections to both FCR and ECR. In the PAS plus contraction condition, the participants were required to attend to a computer screen. This displayed the flexion torque applied at the wrist by the “active arm,” relative to a target corresponding to 20% MVC. In the PAS condition there was no equivalent requirement. It has been demonstrated (Kamke et al., 2012) that the effects of PAS are diminished in conditions of high visual attentional load. It is possible therefore that the attention demands associated with the requirement that the level of wrist flexion torque be monitored continuously prior to the delivery of the peripheral nerve and cortical stimulation, was sufficient to engage neural processes that interfered with those otherwise responsive to the PAS protocol. There are previous reports that when participants attend to visual stimuli located near the limb segment that is the notional target of PAS, the effects of excitability increasing variants of PAS are enhanced – relative to circumstances in which they attend instead to visual stimuli located near the opposite limb (Kamke et al., 2014). In the present study, the focus of visual attention in the combined PAS and contraction condition was upon the display that represented the (contraction) state of the opposite limb. This was located more than a meter from the site of peripheral stimulation. The associated conjecture that in the present case the requirement for attention to be directed toward the visual display may have served to attenuate increases in corticospinal excitability ordinarily realized by PAS (for both FCR and ECR), is consistent with general suppositions concerning the role of attention in regulating the effects of associative plasticity protocols (e.g., Stefan et al., 2004). Nonetheless, it should be noted that differentiating effects of attention on the expression of PAS induced changes in corticospinal excitability are not always obtained (e.g., Dickins et al., 2017).

Neither explanation is, however, able to account for the pattern of outcomes exhibited by the older healthy participants. For this group, both the PAS and the PAS plus contraction interventions gave rise to increases in the amplitude of MEPs recorded in ECR. In neither condition were clear corresponding changes evident for FCR. These features differ from those exhibited by the young healthy participants and the stroke survivors in two respects. Firstly, PAS did not give rise to reliable increases in the excitability of corticospinal projections to the target FCR. Secondly, the application of wrist flexion torque by the opposite limb failed to eliminate the PAS induced effects observed for ECR. Although a recent meta-analysis (Bhandari et al., 2016) suggests that the evidence is not yet definitive, a number of reports have indicated that the extent to which associative stimulation protocols give rise to changes in corticospinal excitability diminishes with age (e.g., Müller-Dahlhaus et al., 2008; Fathi et al., 2010). To the extent that the effects of the specific PAS protocol used in the present study were more readily obtained in ECR than in FCR, it might be supposed that an overall diminution of response in the older participants would account for the failure to observe changes in the state of projections to FCR. This not, however, supported by the fact that with respect to ECR, the magnitude of the effect size corresponding to the change in MEP amplitude induced by PAS was essentially equivalent for the young (*f* = 0.32) and older (*f* = 0.33) healthy adults. It is now widely recognized that there are high levels of variability in both young and older participants (Müller-Dahlhaus et al., 2008), and indeed in terms of the response to PAS more generally (e.g., Guerra et al., 2017). It may simply have been the case that in this relatively small sample of older participants (*n* = 13), the pattern of outcomes evident for the young cohort and for the stroke survivors was simply not manifested reliably across individuals. This factor may similarly account for the observation that wrist flexion contractions performed by the opposite limb did not diminish the PAS induced effects elicited in ECR to the same extent as in the other groups.

There were a number of limitations to the present study. Perhaps most obviously, relatively small groups of participants were engaged. With respect to the stroke survivors in particular, the impact of this limitation may have been compounded further by the heterogeneity of the brain insults that had been sustained. Of the 10 individuals (from more than 100 screened) who participated, three had lesions restricted to cortex, five had lesions restricted to sub-cortical regions, and two had experienced damage to both cortical and sub-cortical regions. The period of time that had elapsed since the stroke ranged from 8 months to over 13 years (median = 3.2 years). On the basis of the Fugl-Meyer Assessment of the Upper Extremity (FMA-UE), all could be categorized as exhibiting “mild impairment” (Woytowicz et al., 2017), since they presented with scores (for the impaired limb) in the range 43–65 (median = 55). In light of this heterogeneity, it is perhaps even more remarkable that the pattern of response to PAS exhibited by the stroke survivors resembled so closely that obtained for the young healthy participants. Necessarily, in any further extension of this line of enquiry, it would be advantageous to employ a sample size sufficient to permit the stratification of participants with respect to the factors referred to above. A further shortcoming was the absence of any means of assessing potential functional consequences associated with the changes in corticospinal excitability brought about by PAS (e.g., Palmer et al., 2018). In light of these various limitations, it must be emphasized that the prospective applicability of these methods in the clinic has not been demonstrated. This field is replete with small-scale “proof of concept” studies that are dramatically underpowered. The present study is no exception. Although in some respects the observations made in the current experiments (i.e., attenuation of PAS induced effects for ECR through flexion contractions of the opposite limb) replicate those of Kennedy and Carson (2008), it is not apparent that the effects would have any direct benefit for the stroke survivor. Indeed, a strong argument can be made that interventions of this nature should have as their goal an increase in the excitability of corticospinal projections to the wrist extensor muscles (and diminution of excitability for projections to wrist flexor muscles). A more general point is demonstrated. Many investigations (such as the present study) that aim to evaluate the clinical potential of one or other variant of NIBS, are not able to produce findings that permit translation to the rehabilitation setting.

Since the first formative descriptions of the phenomenon (e.g., Stefan et al., 2000, 2004; Quartarone et al., 2003), it has been widely surmised that: as the effectual inter-stimulus intervals lie within a restricted (milliseconds) range; and the polarity of the induced effects appears to depend on the order of the stimulus-generated cortical events, PAS induces a particular form of long-term potentiation (LTP) and depression (LTD). Specifically, a resemblance to spike-timing dependent plasticity (STDP) – as it has been elaborated in animal models, has been highlighted (e.g., Müller-Dahlhaus et al., 2010). The characteristics of the original defining studies notwithstanding, it has also been reported that qualitatively equivalent outcomes can be obtained using variants of PAS that lack some of the notional prerequisite features. For example, sustained increases in corticospinal excitability can be brought about using pairs of stimuli that cannot be defined in terms of a discrete ISI (e.g., Ridding and Taylor, 2001; McNickle and Carson, 2015). On the basis of such findings, it has been suggested that multiple cellular pathways (i.e., extending beyond those that mediate STDP) are likely to play a role in mediating the LTP- and LTD-type responses brought about by PAS (Carson and Kennedy, 2013; Suppa et al., 2017). In the present study it was demonstrated that contractions performed by the opposite limb, which extended over many tens of milliseconds, and which preceded the cortical stimulus element of the associative protocol by more than 500 ms, engage processes that interact in a systematic fashion with those that otherwise induce increases in corticospinal excitability. The current results therefore add to the corpus of work indicating that associative effects may be manifested at the systems level in human, even when the timing of the contributory elements is not strictly confined (e.g., McNickle and Carson, 2015; Shulga et al., 2016).

In summary, the outcomes of the present study indicate that PAS may be used to increase the excitability of corticospinal projections to a wrist flexor and a wrist extensor muscle in chronic stroke survivors. The results further demonstrate that in chronic stroke survivors (and in young healthy adults), wrist flexion contractions performed by the opposite limb – which trigger the delivery of paired peripheral nerve and cortical (i.e., associative) stimulation, serve to nullify increases in corticospinal excitability otherwise brought about by PAS. It remains to be determined whether this latter effect is mediated by demands upon attention associated with a requirement to produce graded muscle contractions, and/or whether the restriction of the effect to specific muscles depends upon the nature of the PAS protocol that is employed.

## Author Contributions

RC conceived and designed the study, analyzed the data, and had principal responsibility for writing the manuscript. MR collected and analyzed the data, and contributed to the writing of the manuscript.

## Conflict of Interest Statement

The authors declare that the research was conducted in the absence of any commercial or financial relationships that could be construed as a potential conflict of interest.
